# The multiple layers of RNA response in double-strand break repair

**DOI:** 10.1038/s12276-025-01572-4

**Published:** 2025-11-15

**Authors:** Yilin Lu, Francesca Storici, Youngkyu Jeon

**Affiliations:** 1 School of Biological Sciences, Georgia Institute of Technology, Atlanta, GA USA; 2 Molecular Targets Program, Center for Cancer Research, National Cancer Institute, National Institutes of Health, Frederick, MD USA

**Keywords:** Double-strand DNA breaks, Homologous recombination, Non-homologous-end joining

## Abstract

RNA molecules are now recognized as active regulators of DNA double-strand break repair. In end-joining pathways, nascent transcripts promote repair through RNA:DNA hybrids, end bridging and RNA-templated synthesis. In homologous recombination, RNA:DNA hybrids modulate DNA end resection, recruit repair factors and enable RNA-templated repair, with DNA polymerase ζ emerging as a key reverse transcriptase in this process. Transcription at double-strand break sites generates regulatory RNAs that further influence pathway choice and repair fidelity. Long noncoding RNAs, RNA-binding proteins and RNA modifications add additional control layers. Advances in genomic mapping, reporter assays and in vitro methods are now dissecting these complex RNA-mediated processes, although important challenges remain in capturing their full kinetics and contributions. Finally, RNA-templated genome editing platforms, such as prime editing, harness these principles for precise, programmable DNA repair. Together, these findings position RNA as a multifunctional player in genome maintenance and engineering.

## RNA molecules help repair broken DNA

RNA, once thought to only carry genetic information, also helps fix dangerous DNA double-strand breaks. It can hold broken DNA ends together, pair with DNA or even act as a template for repair. Research shows RNA directly or indirectly supports multiple repair pathways, helping maintain genome stability. These insights are also driving new genome-editing tools, such as prime editing, that harness RNA’s repair abilities.

This summary was initially drafted using artificial intelligence, then revised and fact-checked by the author.

## Introduction

### When RNA repairs DNA: the first responder and a multilevel regulator of genome stability

Long regarded as a passive messenger, RNA is now emerging as an active and versatile regulator of DNA double-strand break (DSB) repair. Both preexisting transcripts and damage-induced RNAs rapidly engage DNA lesions, acting as first responders to genomic insults. Yet, RNA’s influence extends beyond the initial response, contributing to DSB repair at multiple levels, through bridging of broken DNA ends, RNA:DNA hybrid formation, RNA-templated synthesis, recruitment and scaffolding of repair factors, modulation of pathway choice and fine-tuning of chromatin environments.

Importantly, transcription is pervasive across eukaryotic genomes, particularly in higher eukaryotes such as humans^1,2^ where even low-level transcription of noncoding and repetitive regions, alongside active gene transcription, ensures that RNA molecules are broadly available to participate in repair at most genomic loci. This widespread transcription provides a rich and dynamic source of RNA that can influence repair outcomes across diverse chromatin contexts.

In this Review, we explore how RNA functions as a structural, informational and regulatory molecule across key DSB repair pathways, including nonhomologous end joining (NHEJ), microhomology-mediated end joining (MMEJ) and homologous recombination (HR), and discuss emerging technologies and applications that are reshaping our understanding of RNA’s role in genome maintenance.

## RNA in end-joining repair of DSBs

### Direct participation of RNA in end joining

The primary mechanism for repairing DNA DSBs in human cells is NHEJ. This pathway is rapidly activated and functions throughout the cell cycle, making it the first line of defense against DSBs. NHEJ involves direct ligation of broken DNA ends without the need for a homologous template, initiated by binding of the Ku70/Ku80 heterodimer and coordinated by DNA-PKcs, XRCC4, Ligase IV and other factors. Although potentially error-prone, NHEJ’s speed and accessibility make it the predominant repair pathway in most conditions, particularly during the G1 phase of the cell cycle^3,4^. An alternative end-joining pathway, MMEJ or Alt-NHEJ, uses short homologous sequences flanking the DSB site to align the broken DNA ends, typically resulting in deletions at the repair junction^5,6^. While RNA was first recognized for its templating role through its capacity to directly transfer genetic information to chromosomal DNA via homologous interactions^7,8^, emerging evidence reveals that RNA can also serve as the initial molecule to engage and guide DSB repair through end-joining mechanisms, including both NHEJ and MMEJ, directly participating in DNA restoration in a sequence-specific manner independent of a DNA template.

**Fig. 1:**
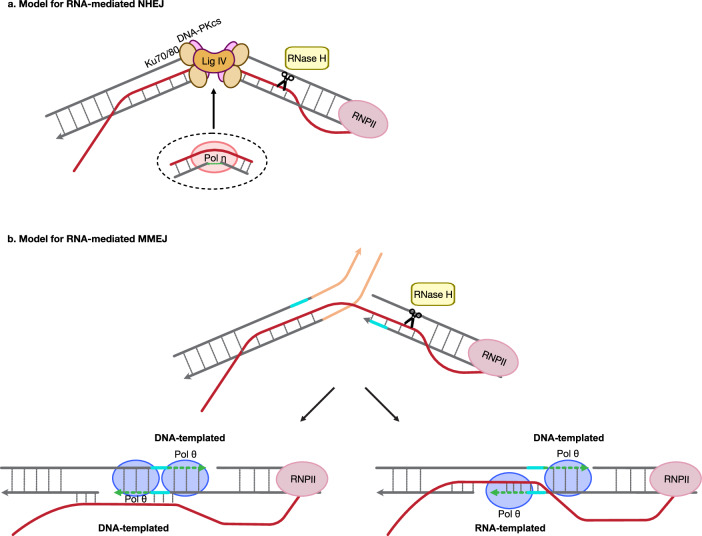
Models for RNA-mediated end-joining repair pathways (R-NHEJ and R-MMEJ) in mammalian cells. **a** Model for R-NHEJ. An endogenous nascent RNA transcript generated from a gene that later experiences a DSB serves as a bridge, facilitating broken-end proximity by forming RNA:DNA hybrids through base complementarity with the DNA ends. Core NHEJ factors, including Ku70/80, DNA-PKcs and Lig IV, then bind to the DNA ends to protect them and initiate downstream repair. DNA ligase mediates ligation, while Pol η (right) may contribute gap filling through its RT activity. **b** An RNA transcript bridges the broken DNA ends via sequence complementarity while skipping intronic sequences (orange). This RNA bridge brings exon-derived microhomologies (blue) into proximity to facilitate MMEJ. After end resection, DNA polymerase θ fills in (dashed green lines) the gaps using DNA templates (left) or potentially both DNA and RNA templates via its RT activity (right). Both R-NHEJ and R-MMEJ occur in cells with wild-type RNase H levels, although overexpression of RNase H1 reduces their efficiency.

The study by Keskin et al.^8^ demonstrated that endogenous nascent transcript RNA sharing homology with DSB ends can mediate HR with chromosomal DNA *in cis* during DSB repair and proposed two mechanisms for this process: an RNA bridging-template mechanism and an RNA extension-template mechanism. The *in cis* component is particularly important, as it opens the possibility that RNA can repair its own broken DNA gene loci. Moreover, this work suggested that RNA may play a ‘simpler’ structural role in DSB repair by bridging and holding the DSB ends in proximity without serving as a template for DNA synthesis. Consistent with a broader role of RNA and transcription-associated processes in DSB repair, Chakraborty et al. demonstrated that, during DSB repair, mammalian NHEJ proteins assemble into a multiprotein complex with RNA polymerase II (Pol II) and preferentially localize to transcribed genes following DSB induction^9^. Depletion of NHEJ factors significantly impaired DSB repair in transcribed but not in nontranscribed genes^9^. In a more recent study, Jeon et al. demonstrated that nascent RNA transcripts can indeed guide the repair of DSBs and double-stranded DNA gaps in both human and yeast cells via RNA-mediated NHEJ (R-NHEJ) in a sequence-dependent manner^10^ (Fig. 1a). Using an engineered reporter system, it was shown that cells expressing either spliced or unspliced RNA transcripts exhibited distinct DNA repair outcomes, depending on the position of complementarity between the RNA sequence and the DNA ends. When the RNA transcript retained complementarity to the very ends of the DSB—such as in cases where the intron was not spliced and the break occurred at the intron–exon junction(s)—the RNA facilitated direct end joining (R-NHEJ). By contrast, when the RNA transcript underwent splicing and the DSB occurred at the intron–exon junction(s), the RNA promoted deletion of the intronic sequence, either through R-NHEJ or RNA-mediated MMEJ (R-MMEJ). RNA:DNA hybrid formation appeared essential for this function, as overexpression of ribonuclease (RNase) H1, an enzyme that degrades RNA within RNA:DNA hybrids^11^, significantly inhibited the repair process. Nonetheless, R-NHEJ and R-MMEJ were observed in both human and yeast cells wild type for RNase H1 and RNase H2, suggesting that the RNA-mediated role in these pathways is rapid, occurring before enzymes such as RNase H1 and H2 can degrade the RNA strands. Importantly, R-NHEJ and R-MMEJ occurred with high efficiency, affecting the efficiencies of NHEJ and MMEJ in a manner dependent on the sequence complementarity between the RNA and the DNA ends^10^. These findings demonstrated that nascent transcript RNA can directly contribute to the sequence-specific reconstitution of broken DNA ends. Mechanistically, R-NHEJ and R-MMEJ processes operate independently of DNA replication across the region experiencing the break and involve both sense and antisense transcripts^10^. Notably, even low levels of transcription were sufficient to promote RNA-mediated repair, with sequence outcomes that mirror the RNA transcript, highlighting an unexpected, broad capacity for RNA transcripts to influence genome stability and modification. Furthermore, the requirement for Ku70, a core component of the NHEJ pathway, for R-NHEJ in yeast cells provides initial evidence that RNA can directly guide canonical end-joining factors by bridging broken DNA ends^10^.

Clearly, further studies are needed to define the key players, mechanisms and biological contexts of R-NHEJ and R-MMEJ. In mammalian cells, DNA polymerase eta (Pol η), traditionally known for its role in translesion synthesis, has also been implicated in RNA-templated synthesis during NHEJ. Chakraborty et al. showed that Pol η possesses reverse transcriptase (RT) activity in vitro and suggested that it plays a critical role in transcription-coupled NHEJ by enabling error-free repair of DSBs using RNA as a template^12^. At DSBs within actively transcribed genes, RNA Pol II assembles a multiprotein complex with PNKP, 53BP1, Ku70, DNA ligase IV, Pol η and nascent RNA transcripts, which localizes to damage sites to facilitate precise, RNA-guided repair^9,12^. These results support the presence of nascent RNA at DSB sites and are consistent with the potential for direct RNA-guided repair via R-NHEJ in transcribed regions. Furthermore, the DNA-dependent protein kinase catalytic subunit (DNA-PKcs) is required for transcription-coupled NHEJ, further linking core NHEJ factors to RNA-guided repair fidelity in transcribed regions^12^. In the context of MMEJ, recent findings indicate that DNA polymerase theta (Pol θ), a key enzyme in MMEJ, also possesses RT activity. Pol θ undergoes a unique structural transformation to accommodate RNA:DNA hybrids within its active site, a property not observed in other DNA polymerases or retroviral RTs^13^. This remarkable structural plasticity enables Pol θ to promote RNA-templated repair, suggesting a potential mechanistic basis for R-MMEJ. Future studies will be required to fully elucidate the physiological relevance of Pol η and Pol θ RT activity in R-NHEJ and R-MMEJ, and to determine whether this RT activity is essential or dispensable for the execution of these RNA-guided repair pathways. It remains to be established under which cellular contexts RNA-templated synthesis contributes to repair outcomes, and how the dynamic interplay between RNA-mediated bridging and RNA-templated synthesis is regulated during DSB repair via end-joining mechanisms.

Together, these findings provide strong evidence that RNA can act both as a structural and as an informational molecule in end-joining repair, challenging the traditional view of RNA as a passive transcript and highlighting its active role in maintaining genome integrity under stress. Based on these insights, we envision an extension of the RNA-bridging model of Jeon et al., wherein RNA:DNA hybrids hold broken DNA ends in proximity and additionally contribute to the recruitment of key NHEJ and MMEJ factors, including DNA polymerases with RT activity, to facilitate end joining (Fig. 1). In R-NHEJ, RNA:DNA hybrids formed at DSB ends promote Ku70 loading, with Pol η and DNA ligase IV mediating precise ligation (Fig. 1a). In R-MMEJ, annealing at microhomologies is supported by the RNA transcript, with DNA synthesis and ligation facilitated by Pol θ and PARP1. Notably, Pol θ possesses RT activity in vitro, raising the possibility that RNA-templated synthesis may contribute to this process (Fig. 1b). These refined models integrate established end-joining factors into an RNA-guided repair framework, highlighting how RNA can directly contribute both structurally and functionally to distinct end-joining pathways.

## RNA-mediated modulation of end-joining pathways

### RNA scaffolding and protein interactions in end-joining regulation

DSB repair pathway choice is influenced by chromatin context, with NHEJ being more active in euchromatin, while MMEJ is preferentially utilized in heterochromatic regions^14^. In addition to direct roles of RNA in end joining, such as RNA-bridging and RNA-templated synthesis, emerging evidence points to more indirect RNA-mediated mechanisms that influence DSB repair. For example, in spinocerebellar ataxia type 3 (SCA3), the RNA-binding protein ATXN3 associates with RNA Pol II and NHEJ core components to facilitate transcription-coupled DSB repair; mutant ATXN3 disrupts this interaction, compromising repair efficiency and promoting disease progression^15^. Similarly, the long noncoding RNA (lncRNA) LINP1 functions as a scaffold to enhance assembly of Ku80 and DNA-PKcs, thereby supporting NHEJ activity. LINP1 expression is modulated by oncogenic EGF signaling, linking RNA-mediated scaffolding to context-specific regulation of DSB repair in cancer^16^. Together, these findings illustrate that RNA can contribute to DSB repair through a continuum of mechanisms, from direct structural and informational roles at DNA ends to indirect scaffolding and regulatory functions mediated by RNA-associated proteins and lncRNAs.

### Regulatory functions of R-loops in NHEJ

R-loops are three-stranded nucleic acid structures composed of an RNA:DNA hybrid and a displaced single-stranded DNA (ssDNA) strand. While traditionally associated with transcriptional regulation and genome instability, R-loops have recently been implicated in the regulation of DNA DSB repair, including NHEJ. At transcriptionally active loci, preexisting transcriptional activity can promote R-loop formation at DSB sites. These R-loops often arise from RNA transcribed before DNA damage, pairing with homologous DNA sequences near the break site^17^. R-loops serve as early regulators of repair pathway choice by physically impeding DNA end resection, through steric inhibition of nucleases such as MRE11 and EXO2. This obstruction favors NHEJ over HR by facilitating the recruitment of end-joining factors and promoting precise repair initiation^17^. Following DSB induction, ssDNA exposed within R-loops is stabilized and protected by RAP80, a component of the BRCA1-A complex^18^. RAP80 prevents excessive nucleolytic degradation by inhibiting CtIP-mediated processing of ssDNA, ensuring that controlled processing of the RNA:DNA hybrid occurs subsequently via RAD52 and XPG. This controlled sequence of events supports precise end joining, mediated by BRCA1, Pol θ and ligases LIG1/3, even in the absence of extensive end resection^18^. These observations underscore a cooperative role between R-loop protection and end-joining fidelity in transcriptionally active regions.

Resolution of R-loops is also critical for effective NHEJ initiation. The exonuclease XRN2 promotes resolution of RNA:DNA hybrids, enabling Ku70 to access and protect DNA ends, thereby facilitating NHEJ^19^. This suggests that timely hybrid resolution is a prerequisite for Ku-mediated end protection and efficient ligation. The R-loop function in DSB repair is further modulated by RNA modifications. The RNA methyltransferase TRDMT1 catalyzes 5-methylcytosine (m⁵C) modification of R-loop RNA, which acts as a suppressive mark to inhibit activation of the alternative MMEJ pathway^20^. This methylation limits PARP1 recruitment and activation, thereby restraining this error-prone repair mechanism and promoting genome integrity at transcriptionally active sites. PARP1 itself plays a complex role in R-loop biology. In the absence of its ADP-ribosylation activity, PARP1 inhibition leads to abnormal R-loop accumulation, unresolved RNA:DNA hybrids and elevated γH2AX signaling, a hallmark of genome instability^21^. Under normal conditions, however, PARP1 is activated by R-loops and contributes to their resolution. PARP1 interacts with the RNA helicase DHX9, a key R-loop processing enzyme involved in transcriptional termination and R-loop removal^22^. This interaction positions PARP1 at the intersection of transcription, R-loop resolution and DSB repair, suggesting that it may integrate R-loop-derived signals to modulate pathway choice and potentially guide MMEJ under stress or pathological conditions.

## RNA in DSB repair via HR

### Direct participation of RNA in the HR pathway

**Fig. 2:**
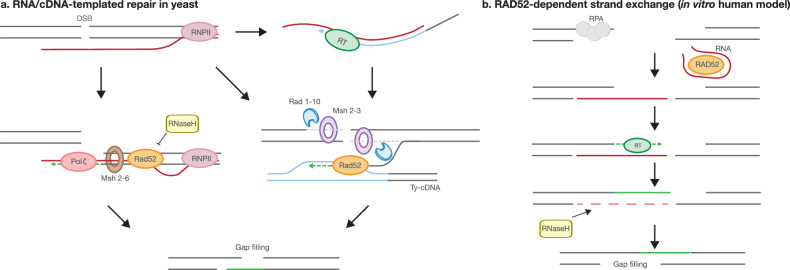
RNA serves as a direct or indirect template to guide DNA DSB repair via HR. **a** In yeast, *cis*-acting RNA transcripts can directly serve as templates for DSB repair (R-TDR) in cells lacking RNase H activity, via an HR mechanism (left). Rad52 facilitates annealing of RNA transcripts to the broken DNA ends through inverse strand exchange. DNA polymerase ζ performs DNA synthesis on the RNA template, extending the 3′ end, followed by single-strand annealing to complete repair. The mismatch repair complex Msh2–Msh6 is also required, probably to correct errors introduced by the low-fidelity activity of Pol ζ. Alternatively, RNA transcripts can be reverse-transcribed by the Ty retrotransposon-encoded RT (Ty-RT) to generate complementary DNA (cDNA), which then serves as a repair template (c-TDR) (right). This pathway requires DNA end resection, with Rad1–Rad10 and Msh2–Msh3 being essential for its execution. **b** In vitro studies have shown that both yeast and human RAD52 promote annealing of RNA templates to resected DNA ends. In reconstitution experiments, the resulting RNA:DNA hybrids can be extended by RTs. After elongation, RNase H cleaves the RNA strand to allow the annealing of the two DNA ends, followed by single-strand annealing to complete repair.

RNA‑templated DSB repair proposes that native RNA itself, without prior cDNA synthesis, can serve as a homologous template for repair. Early studies in budding yeast demonstrated that RNA-containing oligonucleotides or preexisting transcripts of a broken gene can restore genetic information even in the absence of a DNA donor^7,23^. The reaction was markedly stimulated in null mutants of *RNH1*, the gene encoding RNase H1, and of *RNH201*, which encodes the catalytic subunit of RNase H2 (*rnh1*Δ *rnh201*Δ)^7,23^. These results implicate RNA:DNA hybrids as critical repair intermediates and suggest that their persistence is probably required to support extended DNA synthesis. Remarkably, RNA synthesized at the site of the break (*in cis*) served as a much more efficient repair template than RNA provided from elsewhere (in trans), suggesting that immediate availability of the RNA template following lesion induction is critical for efficient repair^7,23^. Biochemical studies revealed that yeast and human RAD52 can bind broken double-stranded DNA and promote inverse strand exchange with homologous single-stranded RNA, stabilizing RNA:DNA hybrids and facilitating polymerase recruitment^24^. The RT function of the yeast retrotransposon Ty is not required for RNA-templated DSB repair (R-TDR)^8,25^. Among the nuclear replicative DNA polymerases in *Saccharomyces cerevisiae*, Pol δ shows limited RT activity and may contribute to R-TDR^7,25^. Further experimental testing in *S. cerevisiae* identified Pol ζ as having the highest RT activity, suggesting it is the primary polymerase mediating R-TDR^25^. Subsequent in vitro studies with purified yeast polymerases confirmed that Pol ζ is the most active RT among yeast DNA polymerases and probably drives R-TDR^26^. The extended 3′-DNA end then completes the repair via single-strand annealing^8,25,27^ (Fig. 2a, left).

The N-terminal half of RAD52 is crucial for both RNA binding and promoting inverse RNA–DNA strand exchange. The C-terminal half, while not directly involved in catalysis, enhances the inverse strand exchange reaction^24,28,29^. Studies using various C-terminal mutants of both yeast and human RAD52 proteins in vitro, as well as expression of these yeast and human RAD52 mutants in yeast cells, have shown that human RAD52 is significantly more active than yeast Rad52 in promoting R-TDR^24^. These results suggest that human RAD52 may play a more prominent and efficient role in R-TDR via HR in mammalian cells, potentially reflecting evolutionary adaptation of this protein to support RNA-guided repair processes in higher eukaryotes.

Reconstitution assays demonstrated that RAD52 directly cooperates with RNA to promote two modes of RNA–DNA repair: an RNA-bridging mechanism that facilitates synapsis and ligation of homologous DNA breaks, and an RNA-templated mechanism that enables reverse transcription-dependent RNA-to-DNA sequence transfer at DNA breaks. RNase H then degrades the RNA strand of the hybrid at the break and RAD52 aligns and anneals the complementary single‑stranded DNA overhangs, completing the single‑strand‑annealing step (Fig. 2b). Both processes are enhanced by transcription of a homologous DNA template in trans, highlighting a direct role for RNA and transcription in coordinating homology-directed repair (HDR) in the absence of a DNA donor^27^. Notably, in postmitotic neurons, RAD52 is specifically recruited to DSBs within actively transcribed regions during G₀/G₁ phases, and inhibition of local transcription significantly reduces RAD52 accumulation^30^. This suggests a critical role for transcription-coupled RNA templates in maintaining genome integrity in nondividing cells, which lack a sister chromatid for classical HR.

Structural and biochemical studies have shown that DNA Pol θ, which is not found in lower eukaryotes like yeast^31^, when bound to RNA:DNA hybrids undergoes a conformational change enabling it to reverse-transcribe RNA:DNA hybrids with velocities and fidelities comparable to retroviral RTs, extending the 3′ DNA end before switching back to canonical DNA synthesis mode^13^. Indeed, human DNA Pol θ has been proposed to play a role in R-TDR^13^. Further supporting the role of RNA templates in DSB repair, in vitro studies have shown that Y-family Pol η can efficiently extend primers annealed to RNA:DNA and DNA:RNA hybrids, even when canonical DNA:DNA duplexes are available, highlighting its intrinsic RT activity^32^. In addition, the retrotransposon LINE-1, which encodes a RT (ORF2p), localizes to Cas9-induced DSBs and inserts cDNA copies of its RNA into the genome, suggesting that LINE-1 RT can be recruited to endogenous lesions in human cells^33^. However, a recent study using complementary fluorescence- and sequencing-based reporter assays demonstrated that DNA Pol θ and LINE-1 RT are not essential for R-TDR in human cells^34^. Instead, RNA-containing oligonucleotides and mRNA were shown to directly serve as templates for DSB repair. Using these assays in combination with a CRISPR–Cas9 genetic screen, the authors identified DNA Pol ζ as the key RT facilitating R-TDR^34^. Furthermore, analysis of cancer genomes revealed whole intron deletions as a mutational signature of R-TDR, highlighting this pathway as an alternative, potentially mutagenic mechanism for repairing DSBs in transcribed genes^34^. These findings further support Pol ζ as the major polymerase mediating R-TDR in yeast, consistent with prior in vivo and in vitro observations for budding yeast Pol ζ (Fig. 2a, left). These results underscore the conserved and central role of Pol ζ in R-TDR across species.

## Indirect roles of RNA in HR pathway

### RNA:DNA hybrids and transcriptional regulation of HR

Transcriptional activity at DSB sites is now recognized as a key regulator of repair pathway choice. Enhancing local transcription or supplying a homologous RNA in trans can increase gene conversion frequencies by ~70% after DSB induction, while RNA Pol II inhibition reduces formation of RPA, RAD51 and BRCA1/2 foci^35–38^. Genome-wide chromatin immunoprecipitation studies show that RAD51 loading depends on the H3K36me3–LEDGF (p75) axis specifically at transcriptionally active loci, a dependency absent in silent chromatin^39^. Conversely, Pol II depletion or elongation arrest shifts repair toward NHEJ^9^. Pol II at the break recruits CtIP, MRE11 and BRCA1, whose activities promote further transcription and stimulate early resection prior to RAD51 filament assembly^40,41^. These findings converge on a model where native transcription coordinates recruitment of cofactors (CtIP and RAD52) to enable HR.

**Fig. 3:**
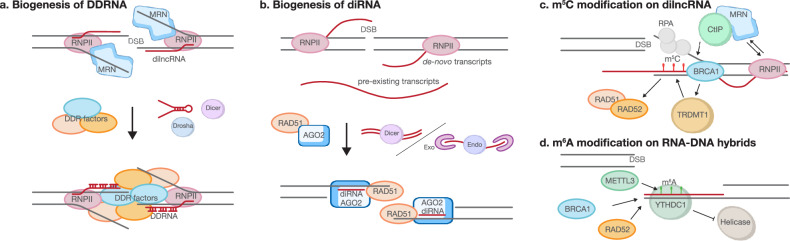
De novo transcription and RNA:DNA hybrid formation following DNA DSB. **a** The MRN complex unwinds DNA at DSB ends, enabling RNA Pol II to initiate transcription at the break site to generate dilncRNAs. Dicer and Drosha process these dilncRNAs to produce DDRNAs, which anneal to residual dilncRNAs and help recruit DNA damage response (DDR) factors. **b** De novo transcription at DSB sites by RNA Pol II generates diRNA precursors, which are processed by Dicer and nucleases to produce diRNAs. AGO2 binds these diRNAs and colocalizes to the break site, where it promotes RAD51 loading. **c** RNA Pol II promotes the recruitment of CtIP and the MRN complex to DSBs, initiating DNA end resection. This resection, in turn, enhances transcription at the break site. The resulting RNA transcripts form RNA:DNA hybrids at the DSB, where TRDMT1-mediated m⁵C modification of the RNA strand facilitates recruitment of RAD51 and RAD52. RNA:DNA hybrids also promote recruitment of BRCA1 to the damage site. **d** METTL3 catalyzes *N*⁶-methyladenosine (m⁶A) modification of RNA:DNA hybrids, stabilizing these hybrids and protecting them from degradation.

Transcription-dependent RNA:DNA hybrids rapidly accumulate at DSB sites, acting as mediators of HR machinery recruitment^42–44^. The MRN complex recruits Pol II to DNA ends, assembling a minimal transcriptional complex that launches bidirectional damage-induced lncRNAs (dilncRNAs) spanning several kilobases around the break^41^. A subset of dilncRNAs is processed cotranscriptionally by Drosha and Dicer into short duplexes, generating DNA damage response RNAs (DDRNAs) that return to the break by base pairing with residual dilncRNA scaffolds^45–47^ (Fig. 3a). Depletion of Drosha, Dicer or AGO2 abolishes BRCA1 foci without affecting γH2AX, demonstrating that DDRNAs amplify checkpoint signaling and license HR independently of histone modifications^48^. In addition, Dicer or Drosha activity or site-specific synthetic ncRNAs can restore the DNA damage response in RNase-A-treated cells^46^. Similar mechanisms were first described in *Arabidopsis*, where Pol IV/V-derived transcripts (plants) or Pol II-derived pairs (mammals) are converted into 21-nt DSB-induced RNAs (diRNAs)^49–52^. Knocking down diRNA processing factors reduces HR efficiency, as diRNA-bound AGO2 interacts with RAD51 to promote HR^53^ (Fig. 3b). Another study further revealed that RNA polymerase III can transcribe the resected 3′ DNA tail itself, transiently capping it with an RNA:DNA hybrid that protects the overhang from nucleases until RAD51-mediated strand invasion initiates, adding a third transcriptional source of RNA at DSBs^54^.

However, recent work has reported no evidence for de novo RNA polymerase recruitment to DSBs, instead implicating preexisting transcripts that anneal to resected ssDNA following transcriptional stalling^55^. These contrasting findings may reflect biological context, such as cell type or break type, as well as technical variables, including detection sensitivity, temporal resolution and differences in damage induction or chromatin state. Together, the data suggest that RNA at DSBs can arise from multiple sources, whose relative contributions remain to be resolved.

RNA modifications further tune HR accuracy. The RNA strand of the hybrid engages the BRCA1 C-terminal region^56,57^. The RNA methyltransferase TRDMT1 deposits m^5^C on this strand, enhancing RAD52 binding^20^ (Fig. 3c). Simultaneously, ATM-phosphorylated METTL3 adds m^6^A to dilncRNAs, and YTHDC1 protects these modified hybrids from premature unwinding, sustaining BRCA1/RAD52 retention for efficient homology search^58,59^ (Fig. 3d). Conversely, ADAR2-mediated A-to-I editing reduces hybrid stability; loss of ADAR2 hyperstabilizes hybrids, blocks long-range resection and redirects breaks toward error-prone end joining^60^.

To prevent pathological hybrid persistence, the helicase Senataxin is recruited to transcriptionally active DSBs, where it collaborates with BRCA1 to unwind RNA:DNA hybrids and prevent interchromosomal translocations^61,62^. PRMT5-methylated DDX5 provides an additional layer of resolution by displacing RNA from R-loops and handing it to XRN2 for degradation. DDX5 deficiency stabilizes hybrids, causes asymmetric deletions and is synthetically lethal with BRCA2 loss, highlighting a quality-control circuit that clears RNA before DNA synthesis initiates^63^. Collectively, RNA:DNA hybrids form at transcriptionally active DSBs: MRN recruits Pol II and Pol III, which drive transcription of dilncRNAs, processed by Drosha, Dicer and AGO2, chemically modified by TRDMT1, METTL3 and ADAR2, and ultimately cleared by Senataxin and DDX5. Through this regulatory network, dilncRNAs, DDRNAs and diRNAs integrate chromatin state and epitranscriptomic marks to guide pathway choice, toward either high-fidelity HR or mutagenic alternatives, placing RNA at the heart of genome maintenance.

### RNA:DNA hybrids in DNA end-resection dynamics

**Fig. 4:**
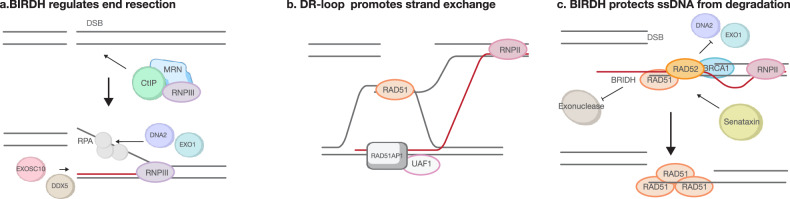
Break-induced RNA:DNA hybrids in regulating end resection, protecting overhang and promoting strand exchange. **a** Following a DSB, CtIP and the MRN complex recruit RNA Pol III to the break site, initiating local transcription. RNA:DNA hybrids formed at the site can be removed by nucleases such as EXOSC10 and DDX5, facilitating RPA binding and enabling subsequent end resection by DNA2 and EXO1. **b** RAD51-associated protein 1 (RAD51AP1), with the assistance of UAF1, promotes the formation of R-loops between the RNA and the homologous DNA donor. This process facilitates RAD51-mediated strand exchange between the ssDNA overhang at the break site and the DNA donor, leading to the formation of a DNA–RNA hybrid displacement loop (DR-loop). **c** Hybrids formed after initial resection protect the ssDNA overhang from degradation and act as a barrier to further resection. Senataxin removes these hybrids, allowing RAD51 loading onto the ssDNA and promoting HR.

RNA:DNA hybrids that accumulate immediately after DSB induction actively shape DNA end processing rather than representing passive byproducts of transcription^42,43^. Genome-wide DRIP-seq and biochemical studies show that diRNAs anneal to newly exposed 3′ ssDNA tails within minutes of resection, forming break-induced hybrids (BIRDHs) that coat both sides of the lesion, particularly in highly transcribed chromatin^43^. These early hybrids protect overhangs from exonucleases and recruit RAD52 and BRCA1; depletion of these factors or forced hybrid dissolution blocks their accumulation and shifts repair toward classical NHEJ^35,42^. Complementing this scaffold, Pol III is recruited by MRN to transcribe the 3′ tail, generating an RNA–DNA cap that further stabilizes the overhang and predisposes the break to HR^54^ (Fig. 4a).

RNA:DNA hybrids also recruit factors that accelerate HR. During S/G2, the RAD51 cofactor RAD51AP1 is brought to transcribed DSBs by SUMO-interacting protein UAF1 and CtIP, facilitating R-loop formation between the resected end and donor duplex DNA, which seeds RAD51-mediated D-loop formation and boosts strand invasion^64^ (Fig. 4b). Loss of CtIP or BRCA1 similarly reduces nascent RNA synthesis at DSBs^40,65^. The RNA:DNA hybrids are dynamic structures^66^. Overexpression of RNase H1 prematurely removes hybrids, triggering excessive bidirectional resection (>160 kb), extensive RPA coating and large deletions within repetitive sequences^67,68^. Thus, RNA:DNA hybrids act as a stop signal to restrain nucleases such as EXO1 and DNA2 and preserve genome stability^69^ (Fig. 4c). HR requires timely hybrid clearance. The nuclear exosome subunit EXOSC10 arrives just before RPA loading, and its depletion blocks RPA assembly and halts resection^37^ (Fig. 4a). Senataxin is recruited as RAD51 filaments form, degrading residual hybrids to promote RAD51 loading and suppress 53BP1, sustaining HR. Cells lacking Senataxin (or *Sen1* in yeast) show reduced HR efficiency and a shift toward end joining^42,61,70^. Senataxin also travels with BLM helicase at actively transcribed breaks^67^. Depletion of BLM reduces RAD51 recruitment and resection but not hybrid levels; it induces PARP1 recruitment and enhances MMEJ. Conversely, loss of Senataxin leads to excess hybrids that trigger BLM/POLD3-dependent repair synthesis, promoting translocations. Depleting POLD3 or BLM reduces these translocations and improves viability in Senataxin-deficient cells^42,71^. PRMT5-methylated DDX5 clears hybrids to allow EXO1 and RPA access; its loss reduces HR by ~70% (ref. ^63^). RNase H2, loaded via PCNA in S phase, trims hybrids that would otherwise interfere with Pol δ, preventing ribonucleotide accumulation and genome instability^72^. RNA-binding proteins such as HNRNPD also contribute by sequestering nascent transcripts away from breaks, preventing hybrid overaccumulation and ensuring proper resection^73^.

Together, these works support a two‑stage, RNA‑driven program regulating DNA‑end resection^42,43,74^. In stage 1, nascent 3′ overhangs are transiently coated with RNA:DNA hybrids formed with diRNAs, which shield ssDNA and recruit RAD52, BRCA1, and RAD51 to establish an HR-competent platform (Fig. 4c). Premature hybrid removal unleashes hyperresection and destabilizes the genome. In stage 2, helicases (Senataxin and DDX5), EXOSC10 and RNase H2 clear excess RNA, enabling RPA loading, RAD51 filament assembly and high-fidelity DNA synthesis.

### RNA as an indirect template for HR through reverse transcription

RNA can supply genetic information indirectly through reverse transcription. In cDNA-templated DSB repair (c-TDR), the cell uses a RT to copy an RNA donor into complementary DNA (cDNA), which is then processed by the HR machinery as a cDNA donor^8,25^. Early studies in *S. cerevisiae* showed that cDNA generated from RNA transcripts could guide precise repair of a site-specific DSBs. This process was blocked by deletion of *SPT3*, a gene required for the RT activity of Ty retrotransposons, implicating Ty-encoded RT as the source of cDNA synthesis^8^ (Fig. 2a, right).

## Approaches to study the role of RNA in DSB repair and genome stability

### Reporter-based assays

A comprehensive understanding of RNA’s role in DSB repair requires assays that capture not only repair outcomes, such as small insertions/deletions and large translocations, but also the local RNA context and repair kinetics. Most conventional reporter assays lack the ability to regulate transcription at the target locus, limiting their utility for dissecting RNA-mediated repair mechanisms. To address these limitations, several groups have engineered reporter constructs incorporating regulatory RNA elements to enable controlled interrogation of RNA’s influence on repair.

In *S. cerevisiae*, an engineered *HIS3* coding sequence containing an artificial intron in the antisense orientation was used to study R-TDR. In this system, only antisense RNA transcripts undergo splicing to remove the intron, thereby generating an intron-free RNA that can serve as a repair template. Successful R-TDR restores an intron-free DNA sequence, enabling cell growth on histidine-free medium^8,25^. A similar strategy was adapted in a human-cell-based plasmid reporter system, where an artificial intron was inserted into the *DsRed* gene. By modifying the intron sequence, Jeon et al. enabled control over splicing and thus created distinct RNA contexts to assess their influence on DSB repair outcomes^10^. In addition, Michelini et al. integrated a tetracycline‑inducible (Tet‑On) promoter upstream of a split GFP reporter (sceGFP), allowing precise control of local transcription levels^36^. Using this system, they demonstrated that transcriptional activation markedly enhances HR at DSBs, confirming that nascent RNA transcripts can promote repair in a transcription‑dependent manner.

### Sequencing-based assays

While reporter assays are powerful, they rely on artificial substrates and have limited cutting site diversity and nuclease choice^75^. To overcome these constraints, sequencing-based approaches have emerged as powerful tools for mapping DSBs and characterizing repair outcomes at nucleotide resolution (Table 1). High‑throughput genome‑wide translocation sequencing (HTGTS) was one of the first assays to capture translocation junctions from an I-SceI ‘bait’ break via circularization or linker ligation, followed by PCR enrichment, enabling profiling of translocations and CRISPR off-target effects^76^. Linear amplification-mediated HTGTS (LAM-HTGTS) and primer-extension-mediated sequencing (PEM-seq) subsequently increased sensitivity more than 10-fold through biotinylated primer enrichment and random molecular barcodes^77,78^. GUIDE-seq and IDLV-seq introduced an alternative strategy by integrating a short DNA template at DSB sites via NHEJ, followed by sequencing of flanking regions to detect indels at frequencies as low as ~0.12%. However, their sensitivity is limited by template integration efficiency^79,80^. Uni‑directional targeted sequencing (UDiTaS) uses Tn5-mediated tagmentation to simultaneously fragment and tag target loci, enabling quantification of both small indels and large structural variants in a streamlined workflow^81^. UMI-DSB-seq ligates adapters containing unique molecular identifiers (UMIs) to both unrepaired DSB ends and intact molecules at flanking restriction sites, allowing direct quantification of broken versus repaired molecules for kinetic modeling^82^.

**Table 1:** Summary of sequencing-based methods for mapping DSB repair outcomes and cleavage specificity.

Method	Assay measurements	Advantages	Disadvantages
HTGTS	Repair outcomes, translocations	Nucleotide-level map of translocation partners	Translocation-biased
LAM-HTGTS	Full repair spectrum (indels + translocations)	Tenfold more sensitive than HTGTS, with UMIs to reduce bias	Requires abundant input material
IDLV-seq	Off-targets, indels	Works in primary and quiescent cells	Limited detection frequency, demanding technical expertise and substantial resource requirements
GUIDE-seq	Off-targets, indels	Low false positive rate	Captures only 30–50% breaks (single-stranded oligodeoxynucleotide (dsODN) dependency)
UDiTaS	On-target repair outcomes	Detects <0.1% indels and large structural variation (SVs), single-tube workflow, low DNA input	Target locus only; needs custom Tn5 reagents
PEM-seq	Full repair spectrum (indels + translocations)	Higher than LAM-HTGTS; captures ultrarare large-scale genomic alterations with UMIs; comprehensive support for the entire analysis pipeline	High DNA input
UMI-DSBseq	Full repair spectrum (unrepaired + indels + translocations)	Kinetic balance of unrepaired versus repaired alleles, time-course ready, optimized for plants	Needs restriction site near cut; locus-specific
Digenome-seq	In vitro cleavage specificity	Simple whole genome sequencing (WGS) pipeline, identify off-target indels at frequencies as low as 0.1%	Requires deep sequencing; many false positive sites
SITE-seq	In vitro cleavage specificity	Low read depth, base-pair resolution mapping	High false positive; needs in-cell follow-up
CIRCLE-seq	In vitro cleavage specificity	180-fold signal-to-noise ratios improvement compared with Digenome-seq, SNP-personalized off-target lists	High false positive; needs in-cell follow-up
CHANGE-seq	In vitro cleavage specificity	Highest in vitro throughput, Tn5-based one-pot workflow; low DNA input	Same false positive caveat as CIRCLE-seq; custom pipeline
BLESS	In vivo cleavage specificity	First nucleotide-resolution in situ mapping, unbiased	High input, high background, requires repair deficient cells and constrained observation period
BLISS	In vivo cleavage specificity	Low input; tissue sections; quantitative	Moderate false positive, demanding technical expertise, requires repair deficient cells and constrained observation period
END-seq	In vivo cleavage specificity	1 break per 10^4^ cells, captures blunt and processed ends	High input, requires repair deficient cells and constrained observation period
DSBCapture	In vivo cleavage specificity	Faster, no plugs	Slightly more shear noise than END-seq, requires repair deficient cells and constrained observation period
i-BLESS	In vivo cleavage specificity	1 break per 10^5^ cells (ultrarare), optimized for small cells	Large starting culture, requires repair deficient cells and constrained observation period
qDSB-seq	In vivo cleavage specificity	Absolute breaks per cell (via spike-in)	Needs restriction spike-ins; wet-lab calibration required
INDUCE-seq	In vivo cleavage specificity	Digital: every read = 1 break, PCR-free; outperforms BLISS, GUIDE-seq, HTGTS	Requires efficient dual-adapter ligation and custom flow cell

These methodologies enable investigators to analyze repair outcomes, including indels, translocations and unrepaired ends, and to assess cleavage efficiency and off-target activity through both in vivo and in vitro assays.

### In vitro screening assays

In vitro assays remain indispensable for evaluating nuclease cleavage efficiency, profiling off-target activity and benchmarking engineered variants in a cell-free context. Digenome-seq performs in vitro Cas9 digestion of purified genomic DNA, followed by whole‑genome sequencing to detect characteristic 5′ ends at cleavage sites^83^. It can detect off‑target indels at frequencies as low as 0.1%, offering greater sensitivity than many cell‑based assays^84^. SITE‑seq further enriches adapter‑tagged DNA ends from RNA-guided Endonucleases (RGEN)‑digested genomic DNA via biotinylation and streptavidin capture, achieving base‑pair resolution with modest sequencing depth (~0.6–2.5 million reads per human genome)^85^. However, only ~10% of in-vitro-identified sites are edited in vivo, reflecting limited predictive value. CIRCLE‑seq refines cell‑free profiling by circularizing sheared DNA before Cas9 treatment, eliminating uncut background and boosting signal‑to‑noise up to 180-fold over Digenome‑seq^86^. CHANGE-seq further simplifies the workflow with Tn5-mediated tagmentation to generate circular DNA libraries, increasing sensitivity while reducing DNA input and processing steps^87^.

### In vivo chromatin-based mapping

Several sequencing-based assays now enable in vivo DSB mapping in native chromatin, with reduced background but lower sensitivity compared with cell-free methods^78^. Breaks Labeling, Enrichment on Streptavidin and Sequencing (BLESS) was the first in situ ligation-based method, attaching biotinylated linkers to DSB ends in fixed nuclei to achieve base-pair resolution of endogenous and induced breaks^88^. BLISS, END-seq and DSBCapture further improved sensitivity and end-resection profiling through enhanced genomic DNA handling and addition of UMIs (~1 DSB per 10^4^ cells)^89–91^. i-BLESS was optimized for small cells such as yeast, achieving ultrahigh sensitivity (~1 DSB per 10^5^ cells)^92^. To enable absolute DSB quantification, qDSB-seq adds a calibrated restriction enzyme spike-in to the i-BLESS workflow, allowing normalization of endogenous break counts per cell^93^. Most recently, INDUCE‑seq uses in situ ligation of full sequencing adapters to DSB ends, followed by direct flow‑cell enrichment without PCR amplification, providing a true digital readout with high sensitivity^94^.

### Leveraging these techniques to study RNA effects on DSB repair

By combining these techniques with transcriptional perturbations, such as transcription inhibitors, RNase H overexpression or RNA metabolism mutants, and sampling at multiple time points after DSB induction, researchers can compare repair kinetics under RNA-rich versus RNA-depleted conditions. END-seq can profile end resection dynamics by quantifying single-stranded overhangs, enabling assessment of how transcription influences resection length. Targeted sequencing assays such as PEM‑seq can catalog repair byproducts, including small indels, microhomology-mediated events and large chromosomal rearrangements, under different RNA conditions. However, because NHEJ completes rapidly after break induction, assays such as END-seq and PEM-seq cannot fully resolve immediate cleavage dynamics or distinguish uncut templates from error-free repair products. To address this, in vitro assays such as SITE-seq or CIRCLE-seq can screen locus-specific cleavage efficiency without interference from end-joining. UMI-DSBseq can be applied at reporter loci to model the ratio of unrepaired to repaired molecules over time, directly linking local RNA availability to repair speed and fidelity.

With the integration of transcription-modulating strategies and advanced sequencing techniques, researchers can now begin to dissect the complex interplay between RNA and DSB repair outcomes across diverse genomic and chromatin contexts.

## RNA-based biotechnology for genome engineering

### RNA-templated editing via prime editing

Prime editing is a CRISPR-based genome engineering system that enables precise insertion, deletion and base substitution without inducing DSBs or requiring donor DNA templates^95^. This system relies on a fusion of Cas9 nickase with an RT, guided by a prime editing guide RNA (pegRNA). The pegRNA combines a standard single guide RNA (sgRNA) extended with an RNA template and a primer-binding site. Upon target recognition, the Cas9 nickase introduces a single-stranded nick, and the pegRNA anneals to the exposed DNA strand, allowing the RT to synthesize the desired sequence directly from the RNA template.

Using RNA as a repair template offers several advantages over DNA-based HDR donors (Table 2). The pegRNA integrates both guiding and templating functions into a single molecule, streamlining delivery and reducing off-target editing risks from component dissociation. In addition, the transient nature of RNA lowers the risk of insertional mutagenesis and immune responses associated with exogenous DNA. Notably, RNA-templated editing is effective in nondividing cells, where traditional HDR is inefficient.

**Table 2:** Advantages of using RNA templates over DNA templates for genome editing.

Advantage	Description
Combined guide and template improving specificity	The pegRNA integrates both guiding (targeting) and templating functions in a single RNA molecule, simplifying delivery and minimizing the risk of off-target editing due to physical dissociation of separate guide and donor components.
Transient nature	RNA degrades naturally, reducing long-term risks such as persistent expression with off-target editing and integration into the genome.
Lower immunogenicity	RNA typically elicits weaker innate immune responses than exogenous DNA donors.
Activity in nondividing cells	RNA-templated editing functions independently of the cell cycle, enabling edits in nondividing cells where HDR is inefficient.

The original prime editor, PE1, has since been optimized for greater efficiency. PE2 incorporates an engineered RT with improved thermostability, processivity and RNA:DNA hybrid affinity^95^. PE3 introduces a second nick on the nonedited strand to bias repair toward the edited sequence via mismatch repair^95^. PE4 and PE5 combine PE2 or PE3 with a dominant-negative MLH1 mutant to further favor desired editing outcomes^96^. PEmax, an optimized PE2 variant, includes a codon-optimized RT, additional nuclear localization signals and enhanced Cas9 activity, substantially improving editing performance^96^. More recently, compact and efficient prime editors (PE6) have been developed through phage-assisted evolution and RT engineering to enhance functionality and enable delivery in size-constrained systems^97^.

Stabilizing the pegRNA itself has also been critical for improving editing outcomes. Insertion of a structured RNA pseudoknot at the 3′ end produces an engineered pegRNA that resists degradation and enhances editing efficiency^98^. In addition, CRISPR interference screening identified the endogenous La protein, a small RNA-binding factor, that protects the 3′ end of pegRNAs from degradation by exonucleases. Fusion of La to the C terminus of PEmax led to the development of PE7, which further improves pegRNA stability and editing efficiency^99^.

Despite these advances, delivering prime editors for therapeutic applications remains a significant challenge, largely due to their size exceeding the packaging capacity of adeno-associated viruses (AAVs)^100–102^. To address this, dual-AAV systems have been developed to split and reassemble the PE components in vivo^97,103–105^. While lentiviral and adenoviral vectors offer larger packaging capacities, they also present limitations: lentiviral vectors pose risks of genomic integration, long-term expression and immunogenicity^106,107^; adenoviral vectors can elicit strong immune responses, particularly against Cas9, potentially causing cytotoxicity^108^. As an alternative, lipid nanoparticles enable transient delivery of mRNA or ribonucleoprotein forms of prime editors with lower immunogenicity and reduced off-target risks. However, current lipid nanoparticle applications are primarily limited to liver targeting, and extending their delivery to other tissues remains an area for further development^109^.

### Emerging questions and future directions

RNA is now recognized as a versatile and multilayered regulator of DNA DSB repair, contributing across diverse repair mechanisms, including NHEJ, MMEJ and HR. Acting as both a first responder and a persistent participant, RNA functions as a structural scaffold, repair template and regulatory signal within these pathways. Yet, many important questions and technical challenges remain to be addressed.

The architecture of eukaryotic genes strongly influences RNA’s potential roles in repair. In higher eukaryotes such as humans, introns account for 90–95% of a typical gene’s sequence, while exons (coding + untranslated regions) represent only ~5–10% (ref. ^110^). As a result, RNA transcripts undergo extensive splicing and alternative splicing, generating RNA populations with variable sequence content. How this dynamic RNA landscape influences repair outcomes, through the formation of RNA:DNA hybrids or the availability of RNA templates, probably depends on the location of DSBs relative to exonic, intronic or spliced regions and remains an important area for future study.

Importantly, even repetitive genomic regions such as telomeres are transcribed^111^, producing RNAs that may impact repair at these fragile loci. Understanding how RNA functions in the repair of repetitive DNA, heterochromatin and other difficult-to-repair regions will be critical for mapping the full scope of RNA’s contribution to genome stability.

While advanced genomic, imaging and biochemical approaches are providing unprecedented insights into RNA-mediated DSB repair, important technical limitations remain. Because some RNA-driven repair processes, such as RNA bridging or RNA-templated end joining, can occur rapidly after break induction, certain DSBs may be repaired and, in some contexts, subject to recutting or multiple repair cycles before mapping or outcome assays are performed. As a result, the observed repair profiles may underestimate or obscure the initial contribution of RNA-dependent mechanisms. This presents a key challenge for accurately capturing the kinetics and full contribution of RNA to DSB repair, particularly for fast or transient events.

Future research will require the development of assays with higher temporal resolution, integration of nascent transcription profiling, and improved live-cell imaging to distinguish RNA-driven repair from later or secondary processes. Key questions include: What determines when RNA acts as a structural versus a templating molecule at DSBs? How does splicing influence the sequence specificity of RNA-guided repair? To what extent do RNA-mediated mechanisms contribute to repair in repetitive and heterochromatic regions? How are these processes regulated across different cell types and states? Addressing these questions will not only deepen our understanding of genome biology but also enable novel RNA-guided genome engineering and therapeutic strategies.
